# Long-term adaptive evolution of *Leuconostoc mesenteroides* for enhancement of lactic acid tolerance and production

**DOI:** 10.1186/s13068-016-0662-3

**Published:** 2016-11-09

**Authors:** Si Yeon Ju, Jin Ho Kim, Pyung Cheon Lee

**Affiliations:** 1 Department of Molecular Science and Technology, Ajou University, Woncheon-dong, Yeongtong-gu, Suwon, 443-749 South Korea; 2 Department of Applied Chemistry and Biological Engineering, Ajou University, Woncheon-dong, Yeongtong-gu, Suwon, 443-749 South Korea

**Keywords:** Long-term adaptive evolution, *Leuconostoc mesenteroides*, d-lactic acid, Acid tolerance

## Abstract

**Background:**

Lactic acid has been approved by the United States Food and Drug Administration as Generally Regarded As Safe (GRAS) and is commonly used in the cosmetics, pharmaceutical, and food industries. Applications of lactic acid have also emerged in the plastics industry. Lactic acid bacteria (LAB), such as *Leuconostoc* and *Lactobacillus*, are widely used as lactic acid producers for food-related and biotechnological applications. Nonetheless, industrial mass production of lactic acid in LAB is a challenge mainly because of growth inhibition caused by the end product, lactic acid. Thus, it is important to improve acid tolerance of LAB to achieve balanced cell growth and a high titer of lactic acid. Recently, adaptive evolution has been employed as one of the strategies to improve the fitness and to induce adaptive changes in bacteria under specific growth conditions, such as acid stress.

**Results:**

Wild-type *Leuconostoc mesenteroides* was challenged long term with exogenously supplied lactic acid, whose concentration was increased stepwise (for enhancement of lactic acid tolerance) during 1 year. In the course of the adaptive evolution at 70 g/L lactic acid, three mutants (LMS50, LMS60, and LMS70) showing high specific growth rates and lactic acid production were isolated and characterized. Mutant LMS70, isolated at 70 g/L lactic acid, increased d-lactic acid production up to 76.8 g/L, which was twice that in the wild type (37.8 g/L). Proteomic, genomic, and physiological analyses revealed that several possible factors affected acid tolerance, among which a mutation of ATPase ε subunit (involved in the regulation of intracellular pH) and upregulation of intracellular ammonia, as a buffering system, were confirmed to contribute to the observed enhancement of tolerance and production of d-lactic acid.

**Conclusions:**

During adaptive evolution under lethal stress conditions, the fitness of *L. mesenteroides* gradually increased to accumulate beneficial mutations according to the stress level. The enhancement of acid tolerance in the mutants contributed to increased production of d-lactic acid. The observed genetic and physiological changes may systemically help remove protons and retain viability at high lactic acid concentrations.

**Electronic supplementary material:**

The online version of this article (doi:10.1186/s13068-016-0662-3) contains supplementary material, which is available to authorized users.

## Background

Lactic acid, or 2-hydroxypropanoic acid, received the US FDA status Generally Regarded As Safe (GRAS) and is used in the cosmetics, pharmaceutical, and food industries. Recently, applications of lactic acid have also emerged in the plastics industry. Numerous studies on lactic acid have been conducted because it is a major raw material for the production of poly-lactic acid (PLA), which is a biodegradable environmentally friendly polymer [1, 2]. Lactic acid has two enantiomers (l-lactic acid and d-lactic acid according to its structure), and there are three types of PLA: optically active l- and d-lactic acids and the racemate. PLA with a high melting point and high crystallinity can be obtained from either the optically pure l- or d-lactic acid isomers, but not from a racemic mixture of the two isomers. Besides, stereocomplex formation among enantiomeric PLA, poly-l-lactic acid, and poly-d-lactic acid enhances mechanical properties, thermal resistance, and hydrolysis resistance [3]. Racemic lactic acid is always produced during chemical synthesis from petrochemical resources, and optically pure l-lactic or d-lactic acid can be obtained by microbial fermentation [4, 5]. Therefore, the selection and characterization of lactic acid bacteria (LAB) that produce large amounts of optically pure lactic acid would be worthwhile. LAB, such as *Lactococcus*, *Lactobacillus*, *Leuconostoc*, *Pediococcus*, *Oenococcus*, and *Streptococcus*, are widely used as lactic acid producers for food-related and biotechnological applications [6]. *Leuconostoc* strains produce d-lactic acid of relatively high optical purity and titer [7]. Recently, metabolic engineering of *Leuconostoc* was used to produce d-lactic acid via overexpression of d-lactic acid dehydrogenase (L-LDH) [8]. However, there are few reports about the metabolic engineering of *Leuconostoc* to enhance the production of d-lactic acid [7].

The ability of organic acids to interfere with microbial vital functions poses a challenge for the microbial production of these compounds at high concentrations to enable an economically viable process [9]. The lactic acid produced by LAB also affects viability of these bacteria owing to the growth inhibition caused by the end product, lactic acid. During fermentation, the growth of LAB is accompanied by lactic acid production leading to acidification of the medium, arrest of cell growth, and possibly cell death due to the entry of the undissociated form of lactic acid into the cytoplasm via simple diffusion [10]. This diffusion of the undissociated form generally follows Overton’s rule, i.e., membrane permeability is a function of molecular hydrophobicity because the cell membranes are composed of lipid domains, which mediate the transport of hydrophobic molecules, and protein pores, which transport hydrophilic molecules [9, 11]. Consequently, dissociation of the lactic acid entering the cells leads to a decline of intracellular pH, and this acidification causes denaturing of essential enzymes, interferes with nutrient transport [12], and damages the cell membrane [9] and DNA via removal of the purine bases [13, 14]. Furthermore, accumulation of anions as a result of the dissociation changes the cell turgor [15] and disrupts key amino acid pools [16]. In response to acid stress, LAB have developed stress-sensing systems such as two-component signaling systems (TCSSes) and can utilize numerous mechanisms to withstand harsh conditions and sudden environmental changes [17]. Some studies have shown that the acid tolerance response (ATR) generally involves the intracellular pH homeostasis via upregulation of proton-pumping F_0_F_1_ ATPase and the production of alkali by arginine deaminase (ADI) or glutamate decarboxylase (GAD) systems [17, 18], alterations of cell membrane functionality, and upregulation of stress response proteins [19–21]. On the other hand, the mechanism of acid tolerance in LAB has not yet been fully elucidated.

Maintaining resistance against acid stressors is vital for the industrial applications of LAB. In this regard, many effective strategies and new protectants have been developed to enhance the functionality of LAB [22]. Recently, adaptive evolution has been used as one of the strategies to gain insight into the basic mechanisms of molecular evolution, resulting in improvements in the fitness and adaptive changes that accumulate in microbial populations during long-term selection under specific growth conditions, such as acid stress [23–25]. During adaptive evolution, several phenotypes of variants that increase fitness in a stressful environment will arise and compete for “dominance” in the total population [23]. Thus, the improved fitness advantage in the mutant cells can improve their viability under stressful conditions, as compared to wild-type and parent strains. Various approaches in other studies have been attempted to investigate the molecular mechanisms of tolerance in the dominant strain. Generally, omics methods combined with molecular techniques have contributed to the understanding and validation of the molecular mechanisms involved in acid tolerance [26, 27]. Moreover, because of the new technologies, such as massively parallel next-generation sequencing (NGS), the relation between a phenotype and a genotype can be elucidated using whole-genome resequencing. Information about the mechanistically validated effects on acid stress can provide guidance to metabolic engineering strategies and increase microbial robustness [9].

The aims of this study were to develop mutant *L. mesenteroides* strains with enhanced lactic acid tolerance by long-term adaptive evolution with exogenously supplied lactic acid and to analyze alterations in the intracellular microenvironment of mutant *L. mesenteroides* strains.

## Methods

### Bacterial strains, plasmids, and growth conditions

All bacterial strains and plasmids used in this study are presented in Table 1. *L. mesenteroides* subsp. *mesenteroides* KCTC 3718 (Korean Collection for Type Cultures, South Korea) was grown at 30 °C in MRS broth (Difco Laboratories, Detroit, MI). The MRS broth (per L purified water) consists of 10 g proteose peptone, 10 g beef extract, 5 g yeast extract, 20 g dextrose, 1 g polysorbate 80, 5 g sodium acetate, 2 g ammonium citrate, 2.0 g K_2_HPO_4_, 0.1 g MgSO_4_∙7H_2_O, and 0.05 g MnSO_4_∙H_2_O. The wild type and mutants were maintained in a laboratory collection as a glycerol stock at −80 °C and were propagated at 30 °C in MRS broth. Working cultures were prepared from stock cultures through two successive transfers (1% inocula) to MRS broth with incubation at 30 °C for 12 h [7]. *Escherichia coli* XL1-blue strain served as a host for a recombinant plasmid and heterologous expression. *E. coli* was grown in Luria–Bertani (LB) medium at 37 °C with vigorous shaking. Chloramphenicol was added to the medium to give a final concentration of 50 µg/mL for *E. coli*. Erythromycin was added to give a final concentration of 20 µg/mL for *L. mesenteroides*.

**Table 1:** Bacterial strains, plasmids, and primers used in this study

Strains or plasmids	Relevant properties	Source or reference
Bacterial strains		
*Leuconostoc mesenteroides*	KCTC 3718 wild type strain carrying pMBLT03	This study
LMS50	Adapted mutant strain in MRS medium supplemented with 50 g/L lactic acid	This study
LMS60	Adapted mutant strain in MRS medium supplemented with 60 g/L lactic acid	This study
LMS70	Adapted mutant strain in MRS medium supplemented with 70 g/L lactic acid	This study
*Escherichia coli*		
XL1-blue	Cloning host for pSTV28	Stratagene
Plasmids		
pMBLT03	*E. coli*-*Leuconostoc* shuttle vector containing *ldhD* gene from *Lactobacillus plantarum*; Em^R^	[9]
pSTV28	P15A derived cloning vector; Cm^R^	TAKARA
pSTV_*atpC*_WT	Expressed *atpC* of *Ln mesenteroides* in *E. coli*; Cm^R^	This study
pSTV_*atpC*_MU	Expressed *atpC* of LMA70 in *E. coli*; Cm^R^	This study
Primers		
*atpC*_WT_F	5′-GGAATTCAGGAGGATTACAAAATGGCAGATGAAACAACAAC-3′	
*atpC*_WT_R	5′-CCCAAGCTTATTTAGCGACCAGATTTCAG-3′	
*atpC*_Mutant_F	5′-GGAATTCAGGAGGATTACAAAATGGCAGATGAAACAACAAC-3′	
*atpC*_Mutant_R	5′-CCCAAGCTTTTTATCGGCTACAAACTGTCAA-3′	

### Strain mutagenesis and screening procedures

Long-term adaptive evolution was conducted to increase lactic acid tolerance of *L. mesenteroides* KCTC 3718. Wild type was adjusted to the stress condition via growth in 120 mL MRS broth containing 30 g/L lactic acid concentration (pH was adjusted to 6.5 with 5 M NaOH) in a 125 mL serum bottle at 200 rpm for 1 week under anaerobic conditions. Next, 1 mL of the cultures was transferred to a fresh MRS medium. After cultivation for 24 h, the cells were spread on MRS agar plates supplemented with 30 g/L lactic acid, 0.05 g/L bromocresol green, and 5% (w/v) CaCO_3_ and then incubated further at 30 °C for 72 h. Mutants were selected based on the size of colonies and a yellow halo zone, and d-lactic acid production of mutants was determined. The mutant with the highest d-lactic acid productivity was repeatedly subjected to the mutagenesis procedure under higher stress conditions (increasing lactic acid concentrations from 40 to 70 g/L). The mutants thus selected on MRS agar containing 50, 60, and 70 g/L lactic acid were named LMS50, LMS60, and LMS70, respectively.

### Analysis of d-lactic acid production

To this end, the selected mutants and parent strains were cultured in 125 mL serum bottles for 1 day, and the cell-free medium was analyzed by high-performance liquid chromatography (HPLC) [28]. d-Lactic acid concentration was determined using an Agilent 1200 Series HPLC system (Agilent Technologies, Santa Clara, USA) equipped with an Aminex HPX-87H column (Bio-Rad, USA) and a refractive index detector (Agilent Technologies, Santa Clara, USA) at a flow rate of 0.7 mL/min and a column temperature of 50 °C, with 4 mM H_2_SO_4_ as the mobile phase. The optical purity of d-lactic acid was determined using a d-Lactic Acid Assay Kit (Megazyme, Ireland).

### Measurement of lactic acid tolerance

The tolerance assays were conducted independently in triplicate in the MRS media containing 0, 15, 30, 45, 60, or 70 g/L lactic acid under anaerobic conditions (pH was adjusted to 6.5 by means of 5 M NaOH). The wild type and mutants were inoculated into 120 mL of the MRS medium in 125 mL serum bottles for the measurement of specific growth rates. Cell growth was monitored every 3 h by the measurement of optical density at 660 nm (OD_660_). Dry cell weight (DCW) was calculated from a curve relating the OD_660_ to DCW: an OD_660_ of 1.0 represented 0.33 g DCW per liter. The initial OD_660_ was set to 0.02, and the specific growth rate (1/h) was calculated according to the formula: *µ* = ln (*X*_2_/*X*_1_)/(*t*_2_ − *t*_1_), where X and t represent the cell concentration (DCW) and the time, respectively.

### Batch fermentation

Batch fermentation of *L. mesenteroides* was performed in a 1.5 L bioreactor (Fermentec, South Korea) with a working volume of 1 L of the MRS medium supplemented with 200 g/L glucose. Seed cultures (100 mL) were prepared in the MRS medium and inoculated into the 1.5 L bioreactor under anaerobic conditions (established by means of N_2_ gas) at 30 °C. The impeller speed was maintained at 200 rpm, and the culture pH of 6.5 was maintained by the automatic addition of 5 M NaOH.

### Two-dimensional (2D) gel electrophoresis and protein identification

Extraction of total protein was carried out by bead beating using 0.4 mm glass beads in a bead beater (Biofact, Korea) for 2 min (alternating pulses: on for 1 min and off for 1 min) at 4 m/s. The protein concentration in each extract was measured using a Qubit 2.0 fluorometer (Invitrogen, USA). After that, 400 μg of a crude protein extract was dissolved in 150 μL of rehydration buffer and was applied to an immobilized pH gradient (IPG) strip with the pH gradient range of 4–7 (GE Healthcare, USA). In the first dimension, isoelectric focusing (IEF) was carried out on an IPGpore, focused for 55,000 V∙h. After IEF, each strip was incubated for 15 min in SDS equilibration buffer [6 M urea, 29.3% glycerol, 75 mM Tris–HCl pH 8.8, 2% SDS, and 0.002% (w/v) bromophenol blue] containing 1% (w/v) DTT and was subsequently incubated with 2.5% (w/v) iodoacetamide [26]. The second dimension was resolved in a 12% polyacrylamide gel in 390 mM Tris–HCl (pH 8.8), 0.1% ammonium persulfate (APS), 0.1% SDS, and 0.04% tetramethylethylenediamine (TEMED). The gels were stained with Coomassie Brilliant Blue R350. Spot analysis was conducted in the ImageMaster 2D Platinum Software (GE Healthcare, USA). The selected proteins were identified by matrix-assisted laser desorption ionization time-of-flight (MALDI-TOF) mass spectrometry. The data files of proteins were analyzed by means of the Mascot bioinformatics search engine (http://www.matrixscience.com) to search the NCBI database.

### Analysis of concentrations of intracellular ATP, NADH, and ammonia

For analysis of intracellular NADH, NAD^+^, ammonia, and ATP, the cells in the log and late exponential phases grown at 30 °C were harvested by centrifugation (12,000×*g*) at 4 °C for 30 min and washed twice with 1 mL of 200 mM cold phosphate buffer (pH 7.4). Next, 20 mg of the cells were sonicated for 6 min at 80% amplitude in a Sonics Vibra-Cell™ sonicator (pulses on for 2 s and off for 10 s). To measure intracellular NADH and NAD^+^, a NAD^+^/NADH Assay Kit (BioAssay Systems, USA) was used. To measure the intracellular ammonia concentration, an Ammonia Assay Kit (BioAssay Systems, USA) was applied. The intracellular ATP concentration was determined by means of an ATP Assay Kit (Abcam, USA).

### Genome resequencing

Whole genomic DNA samples of the wild type and three mutants were extracted by means of the Genomic DNA Kit (Macrogen, Korea). Sequencing of each genomic DNA was carried out using Illumina HiSeq 2000 by the paired-end sequencing technology according to standard Illumina protocols. Complete genome sequence of *L. mesenteroides* ATCC 8293 (GenBank no. NC_008531), a strain equivalent to KCTC 3718 [29], was used as a reference sequence to detect genetic alterations. Conventional PCR and Sanger sequencing service (Macrogen, Korea) were used to confirm the sequence changes observed after Illumina sequencing.

### Computational prediction of 3D structure of the AtpC protein

The 3D structures of the AtpC proteins from the wild type and mutants were predicted by protein folding recognition by means of the Phyre software version 2.0 (http://www.sbg.bio.ic.ac.uk/pyyre2/html). The protein images were visualized in PyMOL, version 1.3.

### Validation of the effect of overexpression of F_0_F_1_ ATPase ε subunit on acid tolerance of *E. coli*

An *atpC* gene encoding F_0_F_1_ ATPase ε subunit of wild-type *L. mesenteroides* and mutant LMS70 strain was amplified by PCR with gene-specific primers from genomic DNA samples and cloned into pSTV28, resulting in vectors pSTV_*atpC*_WT and pSTV_*atpC*_MU, respectively (Table 1). Tolerance of *E. coli* strains harboring pSTVM, pSTV_*atpC*_WT, or pSTV_*atpC*_MU, to acid shock (pH 3.0) was determined as described previously [30]. Cell viability (or acid tolerance) was measured by counting colony-forming units (CFUs) on plates.

## Results

### Adaptive evolution of *L. mesenteroides* in order to enhance acid tolerance and lactic acid production

The adaptive evolution technique was used here to reinforce lactic acid tolerance in d-lactic acid-producing *L. mesenteroides*. Three mutants with enhanced lactic acid tolerance were designated as LMS50, LMS60, and LMS70, where the numbers indicate the concentration of lactic acid present in the selection media. They were isolated after serial transfers in a medium containing progressively increasing concentrations of lactic acid (50, 60, and 70 g/L) during 1 year. First, as an indicator of lactic acid tolerance, specific growth rates of the wild type and three mutants were measured and compared as a function of increasing lactic acid concentrations (up to 70.0 g/L; Table 2 and Additional file 1: Fig. S1). With the increasing lactic acid concentrations, all strains showed a roughly linear decrease in specific growth rates. When the lactic acid concentration above 30.0 g/L was present, the specific growth rate of the wild type significantly decreased as compared to the three mutants, indicating that the mutants gained additional lactic acid tolerance during adaptive evolution.

**Table 2:** Comparison of specific growth rates among the wild type and three mutants in the MRS medium supplemented with varying concentrations of lactic acid

Strains	Specific growth rates (1/h)					
	0 (g/L)	15 (g/L)	30 (g/L)	45 (g/L)	60 (g/L)	70 (g/L)
Wild type	0.35 ± 0.02	0.26 ± 0.01	0.14 ± 0.01	0.08 ± 0.02	0.06 ± 0.02	0.05 ± 0.00
LMS50	0.33 ± 0.01	0.27 ± 0.01	*0.23* ± *0.01* ^a^	*0.16* ± *0.01*	0.10 ± 0.01	0.10 ± 0.01
LMS60	0.38 ± 0.02	0.27 ± 0.01	0.22 ± 0.01	0.15 ± 0.01	0.11 ± 0.02	0.11 ± 0.01
LMS70	0.35 ± 0.01	0.27 ± 0.01	0.18 ± 0.01	0.14 ± 0.02	*0.12* ± *0.01*	*0.12* ± *0.01*

^**a**^Italicsface values represent the highest value at the corresponding lactic acid concentration over 30 g/L

**Fig. 1:**
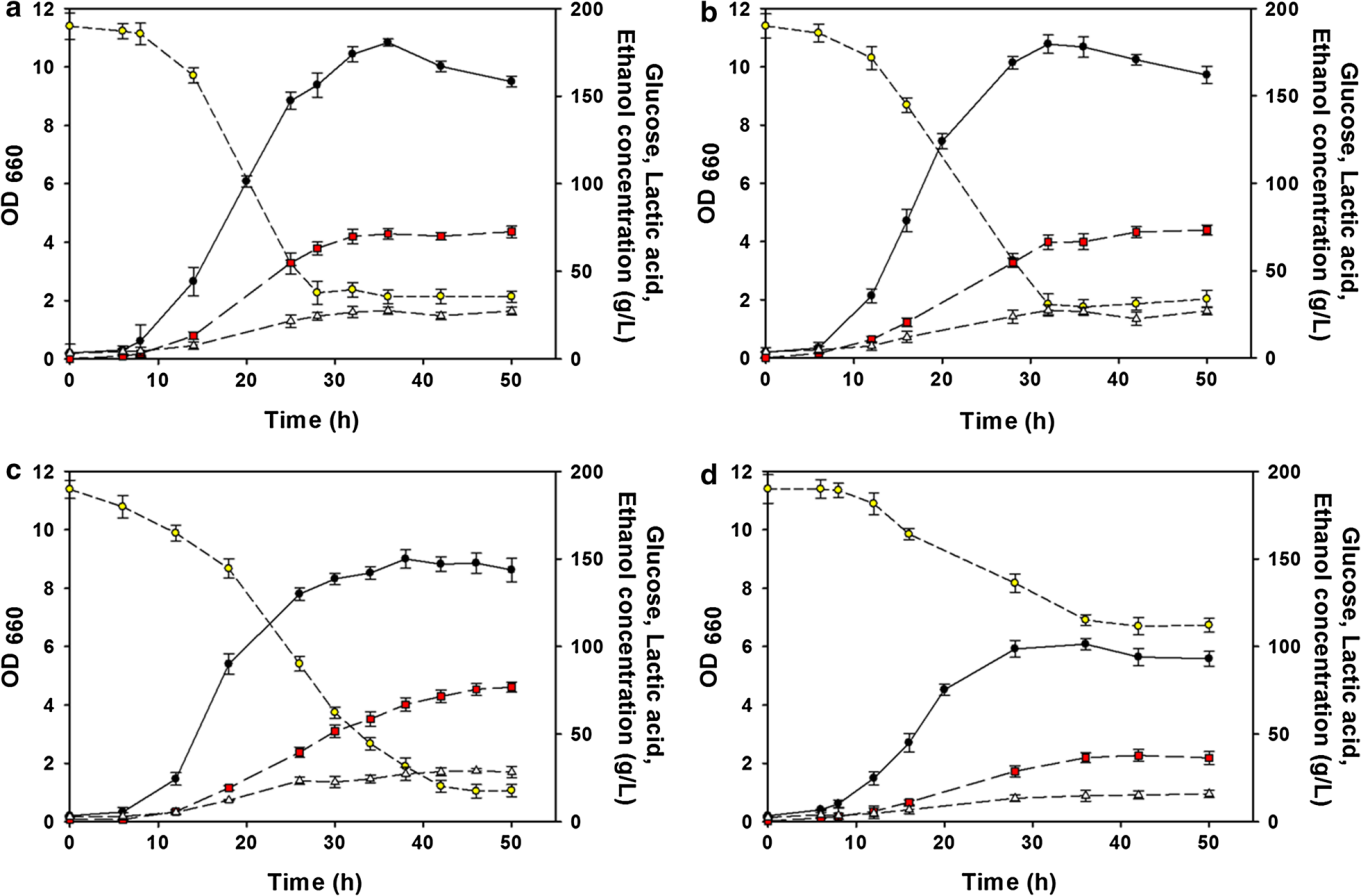
Fermentative production of lactic acid by **a** LMS50, **b** LMS60, **c** LMS70, and **d** the wild type. *Filled circles* represent cell growth; *yellow circles*, glucose; *red squares*, lactic acid; and *open triangles*, ethanol

LMS70 showed higher specific growth rates at >60.0 g/L lactic acid. Next, to evaluate the correlation between d-lactic acid production and enhanced tolerance, the three mutants and wild type were anaerobically cultured in a 1.5 L bioreactor containing the MRS medium supplemented with 200 g/L glucose. LMS50, LMS60, and LMS70 produced 72.6 ± 3.3, 73.2 ± 2.9, and 76.8 ± 2.9 g/L d-lactic acid (Fig. 1a–c), respectively; these figures were ~2.0-fold higher than 37.8 g/L lactic acid produced by the wild type (Fig. 1d). Byproduct ethanol concentrations tended to increase with the increasing lactic acid concentration for all mutants. This result suggests that the mutants that acquired additional lactic acid tolerance could produce more lactic acid.

### Proteomic analysis of the mutant strains

**Fig. 2:**
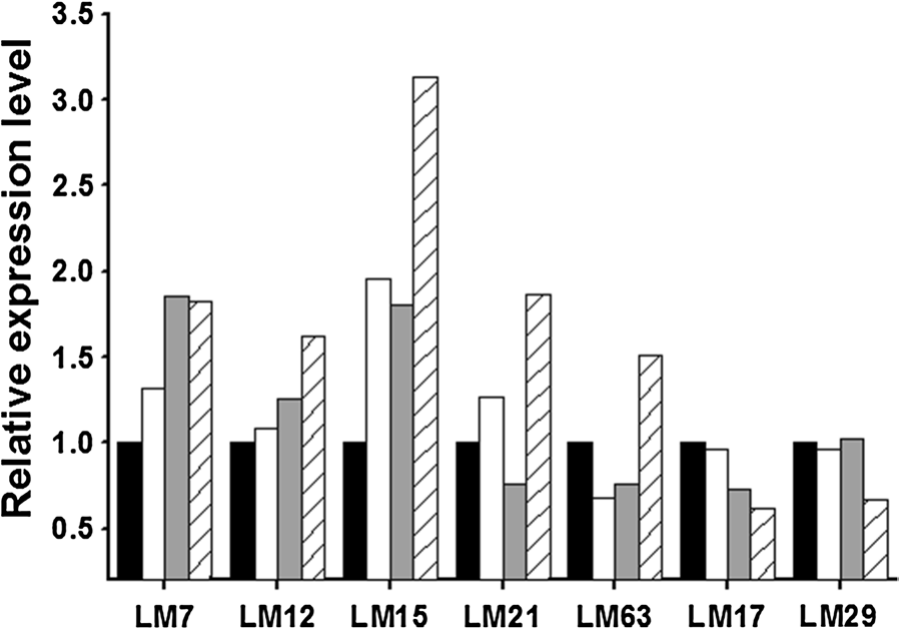
Expression differences for selected spots on 2DE gels of proteomes between the *L. mesenteroides* KCTC 3718 and mutants. Seven spots in 2DE gels of proteomes were selected and subjected to MALDI-TOF analysis. Details on spots are listed in Table 3

To identify proteomic patterns leading to enhanced acid resistance of the mutants, 2D gel electrophoresis (2DE) was carried out. An average of 103 distinct protein spots were observed on 2DE gels. Among them, seven spots (LM7, LM12, LM15, LM17, LM21, LM29, and LM63) showing over twofold changes in intensity (relative to the wild type) were selected and identified using peptide mass fingerprinting (Fig. 2 and Additional file 2: Fig. S2). The five upregulated proteins were identified as translation elongation factor P (EF-P), 5′-methylthioadenosine/S-adenosylhomocysteine nucleosidase 2, phosphoglycerate mutase, phosphomethylpyrimidine kinase, and F_0_F_1_ ATP synthase subunit β. The two downregulated proteins were short-chain alcohol dehydrogenase and chromosome segregation ATPase (Table 3). These differentially expressed proteins could be classified into three categories: cellular metabolism and energy conversion (spots LM12, LM15, LM21, and LM63); DNA replication, RNA synthesis, and translation (spots LM7 and LM29); and hypothetical proteins of unknown function (spot LM17).

**Table 3:** Identified proteins whose expression differed ≥2.0-fold from that in the wild type

COG^a^	Spot no.^b^	Gene	Accession no.	Protein description	Exp.^c^
Translation	LM7	*LEUM_1618*	gi|116618712	Translation elongation factor P (EF-P)	Up
Nucleotide transport and metabolism	LM12	*LEUM_0706*	gi|116617818	5′-methylthioadenosine/S-adenosylhomocysteine nucleosidase	Up
Carbohydrate transport and metabolism	LM15	*LEUM_0251*	gi|116617380	phosphoglycerate mutase	Up
Function unknown	LM17	*LEUM_1148*	gi|504090790	short-chain alcohol dehydrogenase	Down
Coenzyme transport and metabolism	LM21	*LEUM_0143*	gi|116617295	phosphomethylpyrimidine kinase	Up
Cell cycle control	LM29	*LEUM_0346*	gi|116617471	chromosome segregation ATPase	Down
Energy production and conversion	LM63	*LEUM_1869*	gi|488904549	F0F1 ATP synthase subunit beta	Up

^a^ *COG* cluster of orthologous genes

^b^Spot numbers refer to the proteins

^c^ *Exp.*, expression. Upregulation or downregulation of matched proteins

### Analysis of intracellular ATP, ammonia, and NADH concentrations

**Fig. 3:**
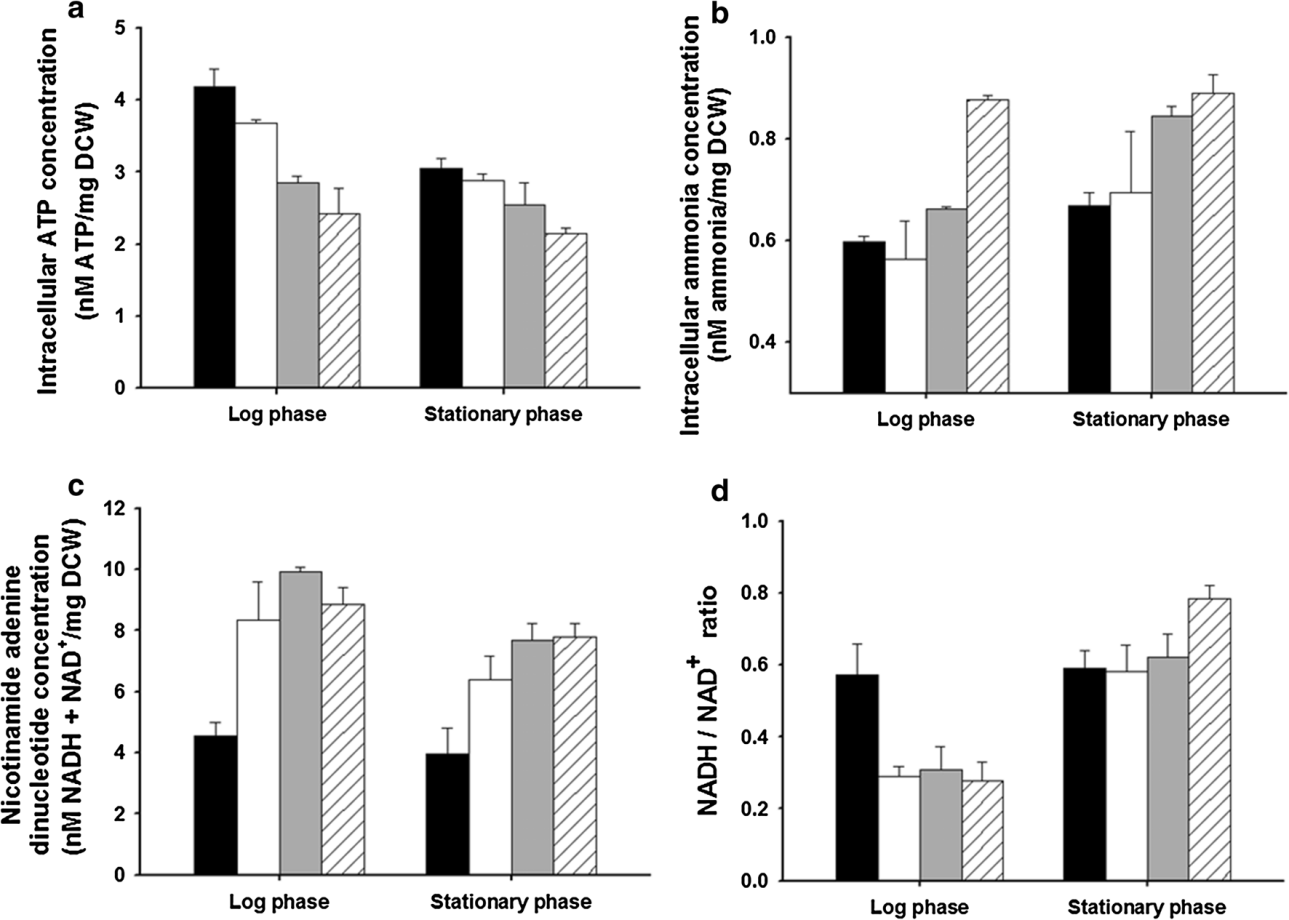
Analysis of microenvironment changes of the wild type (*black bars*), LMS50 (*white bars*), LMS60 (*gray bars*), and LMS70 (*hatched bars*). Amounts of intracellular ATP concentration (**a**), intracellular ammonia concentration (**b**), nicotinamide adenine dinucleotide concentration (**c**), and a NADH/NAD^+^ ratio (**d**) in the wild type and three mutants grown in log and stationary phases, respectively. *Error bars* indicate standard deviations (*n* = 3)

Physiological properties such as intracellular levels of ATP, ammonia, NADH, and NAD^+^ are factors important for acid tolerance [31, 32]. Therefore, intracellular levels of ATP, ammonia, NADH, and NAD^+^ in the wild type and three mutants were analyzed and compared. First, intracellular ATP was extracted from the wild type and three mutants (and quantified) in the log and stationary growth phases. In both log and stationary phases, lower levels of intracellular ATP were detected in the three mutants compared with the wild type (Fig. 3a), but total concentration of ATP in the three mutants and wild type was higher in the log phase than in the stationary phase. In the log phase, a higher ATP level was detected in the wild type (4.19 ± 0.24 [nM ATP]/[mg dry cell weight (DCW)]), followed by LMA50 (3.68 ± 0.05 [nM ATP]/[mg DCW]), LMA60 (2.86 ± 0.08 [nM ATP]/[mg DCW]), and LMA70 (2.42 ± 0.36 [nM ATP]/[mg DCW]). Just as in the log phase, in the stationary phase, a higher ATP level was observed in the wild type (3.05 ± 0.14 [nM ATP]/[mg DCW]), followed by LMA50 (2.88 ± 0.10 [nM ATP]/[mg DCW]), LMA60 (2.54 ± 0.31 [nM ATP]/[mg DCW]), and LMA70 (2.14 ± 0.08 [nM ATP]/[mg DCW]). Next, intracellular ammonia levels in the wild type and three mutants in log and stationary growth phases were quantified. In contrast to intracellular ATP levels, higher amounts of intracellular ammonia were detected in the three mutants in both log and stationary phases compared with the wild type (Fig. 3b). The cellular ammonia concentration in the wild type was found to be 0.60 ± 0.01 nM/(mg DCW) in the log phase and 0.67 ± 0.03 nM/(mg DCW) in the stationary phase. The highest ammonia concentrations (0.88 ± 0.01 nM/[mg DCW] in the log phase and 0.89 ± 0.04 nM/[mg DCW] in the stationary phase) were observed in LMS70, which had better acid tolerance than LMS50 and LMS60 did. Finally, summed amounts of NADH and a NAD^+^ and the NADH/NAD^+^ ratio were measured in the wild type and three mutants in the log and stationary phases. The highest total amount of cellular NADH and NAD^+^ was detected in LMS60 (9.93 ± 0.16 nM/[mg DCW]) in the log phase, followed by LMS70 (8.85 ± 0.56 nM/[mg DCW]), LMS50 (8.36 ± 1.23 nM/[mg DCW]), and the wild type (4.56 ± 0.45 nM/[mg DCW]; Fig. 3c). In the stationary phase, a similar amount of cellular NADH and NAD^+^ was detected in LMS60 (7.69 ± 0.56 nM/[mg DCW]) and LMS70 (7.79 ± 0.44 nM/[mg DCW]). Nonetheless, total amounts of NADH and NAD^+^ (3.96 ± 0.85 nM/[mg DCW]) in the wild type in the stationary phase were 50% lower than those in the mutants. Notably, in both log and stationary phases, the total summed amounts of NADH and NAD^+^ in the three mutants were twofold higher than those in the wild type. In terms of the NADH/NAD^+^ ratio, which indicates cellular redox status, this ratio (0.57 ± 0.09) in the wild type in the log phase was twofold higher than that in the three mutants (0.29 ± 0.03 for LMA50, 0.31 ± 0.06 for LMA60, and 0.28 ± 0.05 for LMA70), but the NADH/NAD^+^ ratio in the wild type increased to 0.59 ± 0.05; this figure was similar to that in LMA50 (0.58 ± 0.07) and LMA60 (0.62 ± 0.06) in the stationary phase (Fig. 3d). The highest NADH/NAD^+^ ratio was observed in LMA70 (0.78 ± 0.04) in the stationary phase.

### Analysis of genetic alterations in the mutants

**Fig. 4:**
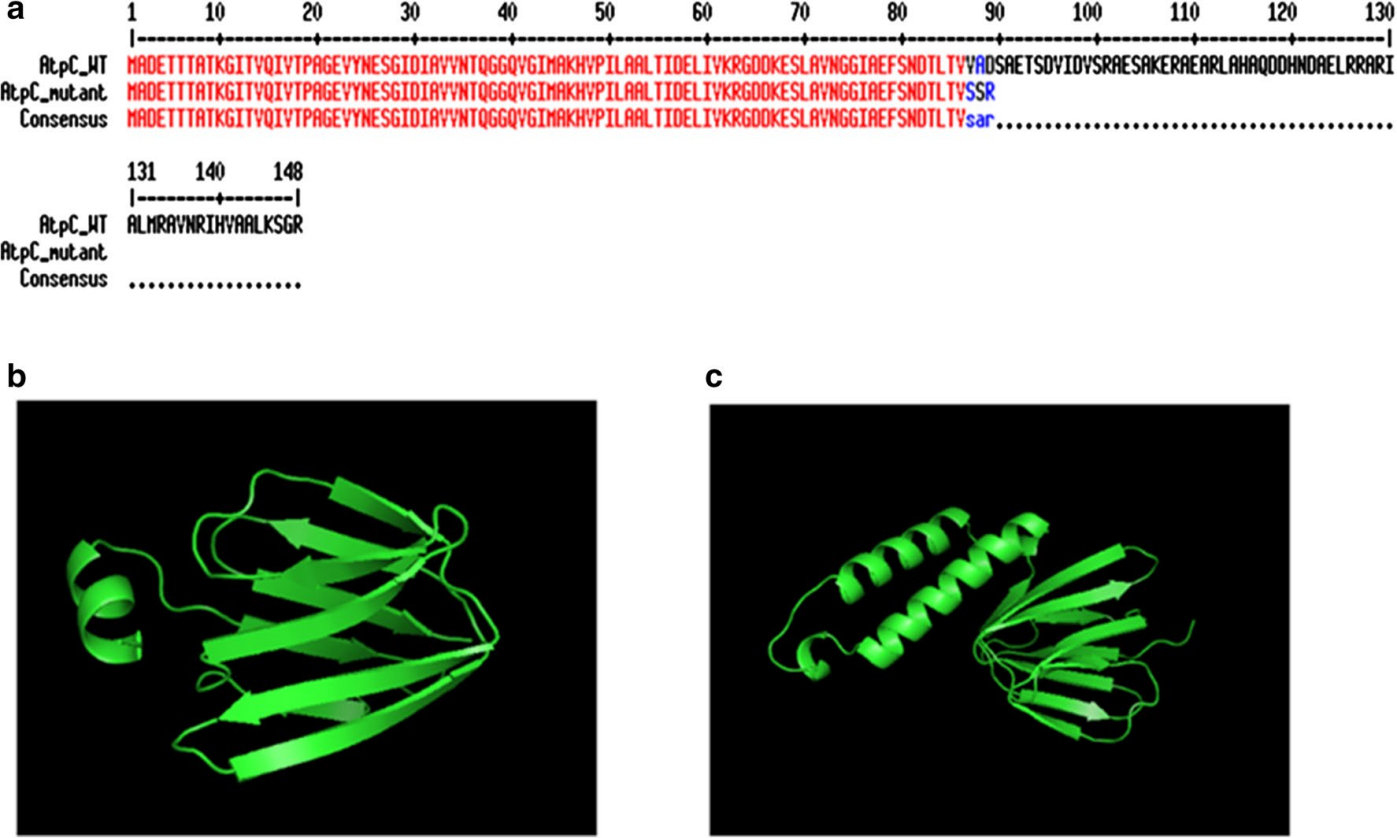
Comparison of mutant and wild-type AtpC proteins. Amino acid sequences of mutant and wild-type AtpC proteins were aligned (**a**). *Red color* represents 100% identity of the mutant and wild type, and *blue color* denotes the altered three amino acid residues (**a**). Computational 3D structures of mutant AtpC protein (**b**) and wild-type AtpC protein (**c**)

To understand how the mutants had evolved to acquire enhanced acid tolerance, the genetic alterations in the genomes of the three mutants were analyzed by the Illumina paired-end sequencing technology. Comparative analysis of incomplete genome sequences of the three mutants with that of the wild type (unpublished data) uncovered a frameshift mutation in the *atpC* gene (encoding F_0_F_1_ ATPase ε subunit: AtpC) in all three mutants. The frameshift mutation was caused by insertion of one nucleotide (A) at position 259, resulting in three amino acid changes (V87S, A88S, and D89R) and a new stop codon at position 270 (Fig. 4a). According to computational modeling, mutant AtpC protein is devoid of the α-helix-loop-α-helix domain (Fig. 4b), which is present at the C terminus of the wild-type AtpC protein (Fig. 4c). Notably, C-terminal structure of the AtpC protein is known to regulate the ATP hydrolysis reaction by restricting rotation of the F_0_F_1_ ATPase rotor [33, 34].

### Effects of the frameshift mutation in the *atp*C gene on acid tolerance of *E. coli*

**Fig. 5:**
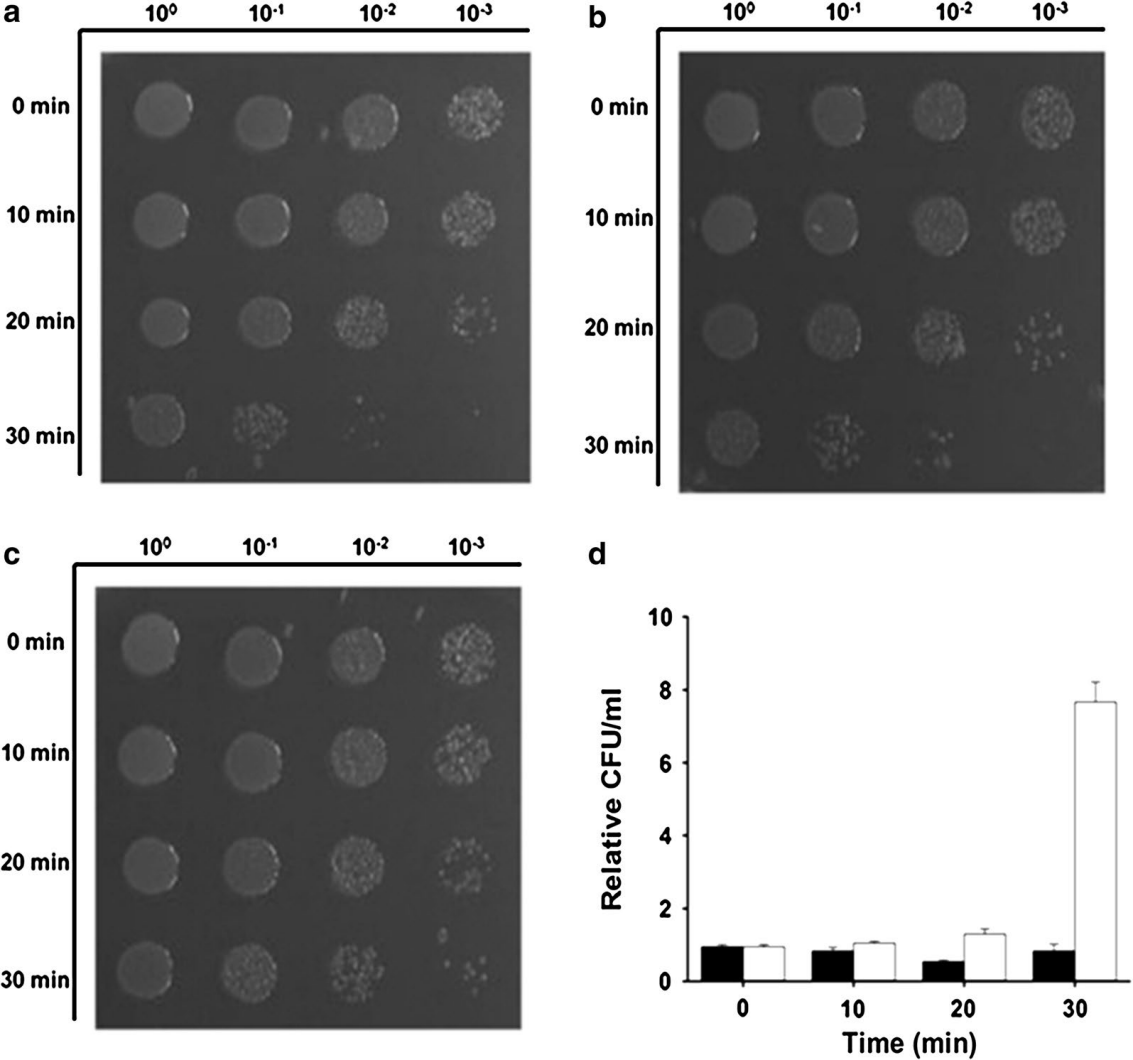
Effects of the overexpression of wild-type and mutant AtpC proteins on the viability of *E. coli* after acid shock. Acid shock and a viability assay were carried out using *E. coli* expressing an empty vector (**a**) or wild-type (**b**) or mutant *atp*C gene (**c**). X- and Y-axes show the dilution factor and treatment time, respectively. Relative survival rates of *E. coli* expressing wild type (*black bars*) and mutant *atp*C gene (*white bars*) are expressed in colony-forming units (CFUs) (**d**). *Error bars* indicate standard deviations (*n* = 4)

To confirm the relation between the frameshift mutation in *atpC* and enhanced acid tolerance of the mutants, acid shock and a viability assay were carried out using *E. coli* expressing an empty vector (Fig. 5a), wild-type (Fig. 5b) gene, or the frameshift mutant *atpC* gene (Fig. 5c). Survival rates of *E. coli* significantly decreased in all *E. coli* after 20 min acid shock (Fig. 5d**)**. Cell viability of *E. coli* expressing the frameshift mutant *atpC* gene increased 7.7-fold after 30 min acid shock, suggesting that the frameshift mutant *atpC* gene exerted a beneficial effect on acid tolerance.

## Discussion

Chemical or physical mutagenesis and adaptive evolution are used for increased production of lactic acid from LABs. Chemical or physical mutagenesis approach is a powerful tool for isolating hyper lactic acid-producing LABs such as mutant *Lactobacillus lactis* NCIM 2368, which was reported to produce up to 110 g/L of d-lactic acid from 150 g cane sugar/L [1]. Adaptive evolution approach has been used to gain insight into the genetic basis and dynamics of adaptation of LAB as well as to isolate hyper lactic acid-producing LABs. In this study, *L. mesenteroides* KCTC 3718 was evolved long term under lethal acid stress to improve acid tolerance and overproduce d-lactic acid. During the adaptive evolution, serial transfers were performed for 1 year to accumulate genetic variants of *L. mesenteroides* under increasing lactic acid stress. It is known that microbial fitness develops rapidly at the first stage of laboratory evolution, and after that, fitness development generally slows down, but genetic mutations steadily accumulate during prolonged selection [23]. This is true for adaptive evolution of *L. mesenteroides*: the mutant LMS50, which was isolated at the earlier evolution stage, showed greater improvement of acid tolerance and lactic acid production in comparison with the previous strain (the wild type). As a fitness parameter, the specific growth rate (µ) of LMS70 increased 2.6-fold as compared to the wild type at a high lactic acid concentration (70 g/L). The d-lactic acid-producing ability of LMS70 was enhanced twofold in comparison with the wild type. On the other hand, the d-lactic acid conversion yield (g-lactic acid/g-glucose) of LMS70 decreased by 3.9% (44.6% in LMS70 vs. 48.5% in the wild type). Chromosome segregation ATPase (LEUM_0346), which expression was downregulated in mutants, was probably involved in controlling cell division under high stress conditions [35].

The alkali production, primarily that of ammonia, is known to serve as a physiological and biological buffer system for acid tolerance by neutralizing excess cellular protons [36]. This notion is also applicable to the three mutants. The ammonia levels in all three mutants increased significantly as compared to the wild type. Redox power plays a pivotal role in lactic acid production because pyruvate is converted to lactic acid with consumption of NADH [37, 38]. Notably, the mutants showed an increased NADH/NAD^+^ ratio in the stationary phase and larger total amounts of NADH and NAD^+^ in both log and stationary phases. This finding implies that in the log phase, the mutant strains had a lower redox ratio because of the vigorous production of lactic acid. Furthermore, 5′-methylthioadenosine/S-adenosylhomocysteine (MTA/SAH) nucleosidase (LEUM_0706), which is one of the five upregulated proteins, may be related to the total amounts of NADH and NAD^+^ in the mutants. We can hypothesize that the overproduction of MTA/SAH nucleosidase can increase adenine pools to fulfill the demand for nicotinamide adenine dinucleotide synthesis in mutants because the above enzyme has dual substrate specificity, regulates intracellular levels of MTA and SAH, and produces adenine for various reactions [35]. Another notable upregulated protein in the mutants is phosphomethylpyrimidine kinase (LEUM_0143). This enzyme is involved in the thiamine synthesis pathway, which is known to contribute to acid tolerance by providing thiamine to acetolactic acid synthase, which contributes to proton consumption [39].

Resequencing of genomes of the mutants identified a frameshift mutation in the *atpC* gene encoding F_0_F_1_ ATPase ε subunit, resulting in the deletion of the C-terminal helix-turn-helix domain and the introduction of three amino acid changes. As validation of the function of the mutant *atpC* gene, acid shock and a viability assay were performed on *E. coli* instead of *L. mesenteroides* because of the lack of well-established genetic tools for *L. mesenteroides*. As expected, the deletion of the C-terminal domain in the AtpC protein enhanced the acid resistance of *E. coli*, indicating that the mutant AtpC protein improved the proton-pumping activity under acidic conditions. A similar result was reported elsewhere; the F_0_F_1_ ATPase containing the ε subunit with a deletion in the C-terminal structure shows a higher rate of ATP hydrolysis than the normal F_0_F_1_ ATPase does [33].

Moreover, these results are consistent with upregulation of the F_0_F_1_ ATPase β subunit (LEUM_1869) and a decline in the intracellular ATP concentrations in the mutants.

The intracellular ATP level in all the mutants tended to depend on acid production (Fig. 3a). This finding suggests that increased proton extrusion activity (conferred by the mutant AtpC protein) and overexpression of F_0_F_1_ ATPase may contribute to increased cell viability in an acid stress environment. Furthermore, upregulated phosphoglycerate mutase (LEUM_0251) probably contributes to smooth ATP production from the carbohydrate metabolism during carbon (glucose) utilization. Generally, LAB under acid stress are known to upregulate the pathway of glucose metabolism (glycolysis) to produce ATP efficiently, as substrate-level phosphorylation rather than oxidative phosphorylation, and this metabolic ploy can fulfill the requirements for ATP hydrolysis of F_0_F_1_ ATPase [26, 38]. This notion is in line with a lower amount of ATP in the mutants here, especially in LMS70, than in the wild type. Notably, compared to other acid-adapted LABs including *L. casei* ATCC 334 [40] and *B. longum* [35], LMS50, LMS60, and LMS70 did not change proteomes involved in amino acid metabolism such as histidine, glutamate, and valine.

LMS70 was evolved further and its offspring were characterized by resequencing, proteomics, and metabolomics. Even though in this study we could not resequence DNA of LMS50, LMS60, and LMS70 because of incomplete genome assembly, the observed combined effect of genetic alterations in the genomes of the three mutants was clearly demonstrated. Further research is needed to fully understand the mechanisms of the acquired acid tolerance of the mutants and to develop robust lactic acid-producing microorganisms.

## Conclusions

Industrial mass production in LAB is a challenge mainly because of growth inhibition caused by the end product, lactic acid. Recently, adaptive evolution has been employed as one of the strategies to improve the fitness and to induce adaptive changes in bacteria under specific growth conditions, such as acid stress. Here, our work presents the first report of the adaptive evolution of d-lactic acid—producing *L. mesenteroides* strain in order to alleviate the effect of acid stress and to enhance d-lactic acid production. We successfully obtained three *L. mesenteroides* mutants (LMS50, LMS60, and LMS70) with improved acid tolerance and d-lactic acid yield. The fitness of the mutants during adaptive evolution gradually accumulated beneficial mutations in lethal acid stress. The increased specific growth rates above 30 g/L lactic acid stress, in which the wild type exhibited relatively low growth rates, were clearly observed in the mutants. Moreover, the enhancement of acid tolerance in the mutants contributed to increased production of d-lactic acid. Especially, the 76.8 ± 2.9 g/L d-lactic acid produced by LMS70, which was isolated in 70 g/L lactic acid stress, revealed about 2.0-fold higher than titer of the wild type. This work provides a feasible approach for strain engineering and advances our understanding of the molecular mechanisms of adaptive evolution, which might provide insight into acid tolerance.

## Additional files

**Additional file 1: Fig. S1.** Growth curves of wild type (black circles), LMS50 (yellow circles), LMS60 (white squares), and LMS70 (red triangles) in MRS media containing increasing lactic acid concentration. Each strain was cultivated in MRS medium without lactic acid (A), and supplemented with lactic acid of 15g/L (B), 30g/L (C) 45g/L (D), 60g/L (E) and 70g/L (F). The cell growth was monitored by optical density (OD) at 660 nm. Error bars indicate standard deviations (*n* = 3).

**Additional file 2: Fig. S2.** 2DE gels of proteomes between wild type (A), LMS50 (B), LMS60 (C) and LMS70 (D).

## Abbreviations

LDH: lactic acid dehydrogenase
HPLC: high-performance liquid chromatography
aa: amino acid residues
2DE: two-dimensional gel electrophoresis
IEF: isoelectric focusing
NGS: next-generation sequencing
