# Supplementary material for: Long-term adaptive evolution of Leuconostoc mesenteroides for enhancement of lactic acid tolerance and production
Source: Biotechnol Biofuels. 2016 Nov 9;9:240. doi: 10.1186/s13068-016-0662-3 (PMC5103595; doi:10.1186/s13068-016-0662-3)

**Figure S1. Growth curves of wild type (black circles), LMS50 (yellow circles), LMS60 (white squares), and LMS70 (red triangles) in MRS media containing increasing lactic acid concentration.** Each strain was cultivated in MRS medium without lactic acid (A), and supplemented with lactic acid of 15g/L (B), 30g/L (C) 45g/L (D), 60g/L (E) and 70g/L (F). The cell growth was monitored by optical density (OD) at 660 nm. Error bars indicate standard deviations (n = 3).


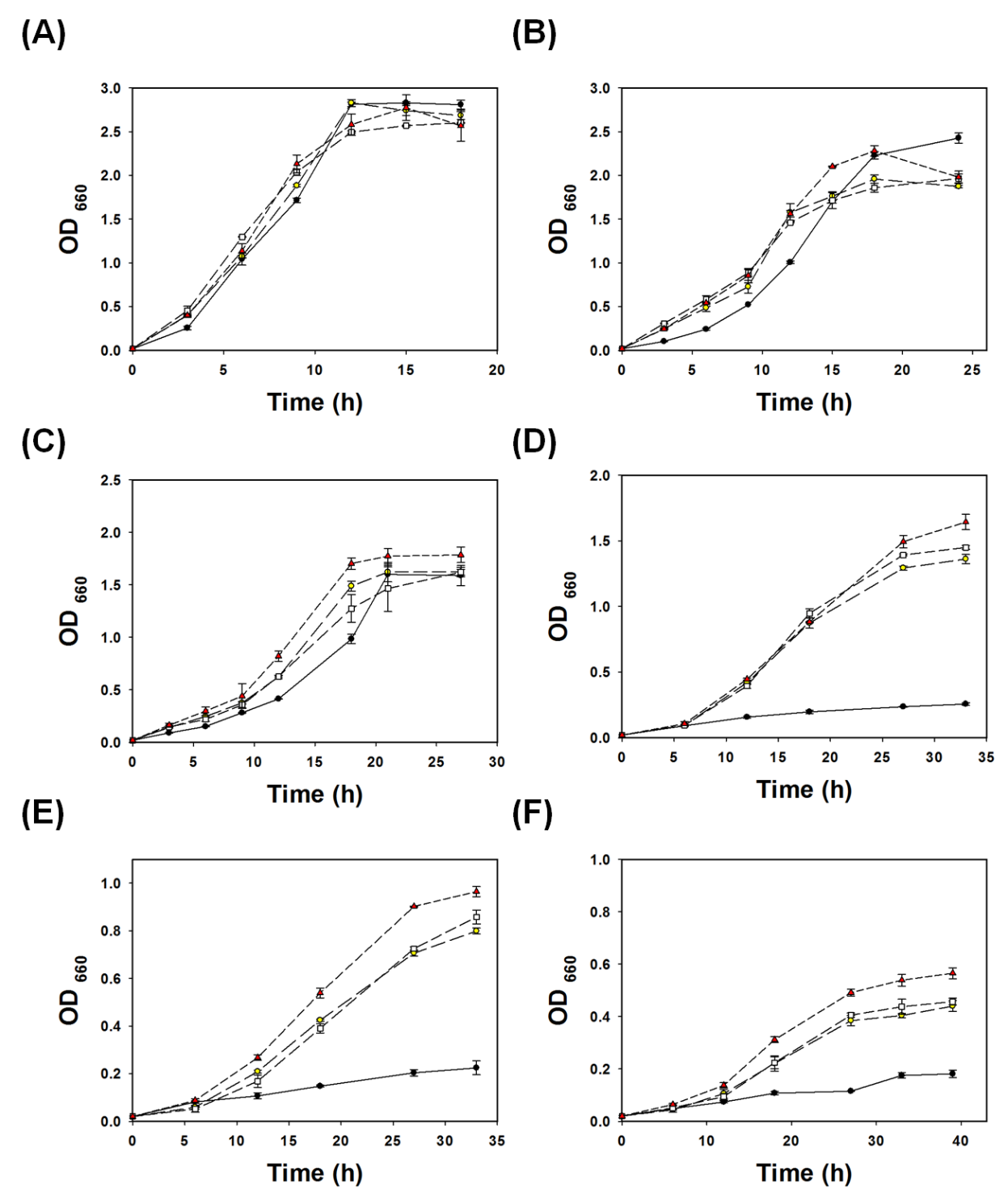

Supplement: Supplementary file 1 — Additional file 1: Fig. S1. Growth curves of wild type (black circles), LMS50 (yellow circles), LMS60 (white squares), and LMS70 (red triangles) in MRS media containing increasing lactic acid concentration. Each strain was cultivated in MRS medium without lactic acid (A), and supplemented with lactic acid of 15g/L (B), 30g/L (C) 45g/L (D), 60g/L (E) and 70g/L (F). The cell growth was monitored by optical density (OD) at 660 nm. Error bars indicate standard deviations (n = 3). [file 13068_2016_662_MOESM1_ESM.docx]
