# Supplementary figures and images for: Long-term adaptive evolution of Leuconostoc mesenteroides for enhancement of lactic acid tolerance and production
Source: Biotechnol Biofuels. 2016 Nov 9;9:240. doi: 10.1186/s13068-016-0662-3 (PMC5103595; doi:10.1186/s13068-016-0662-3)

**Figure S2. 2DE gels of proteomes between wild type (A), LMS50 (B), LMS60 (C) and LMS70 (D).**


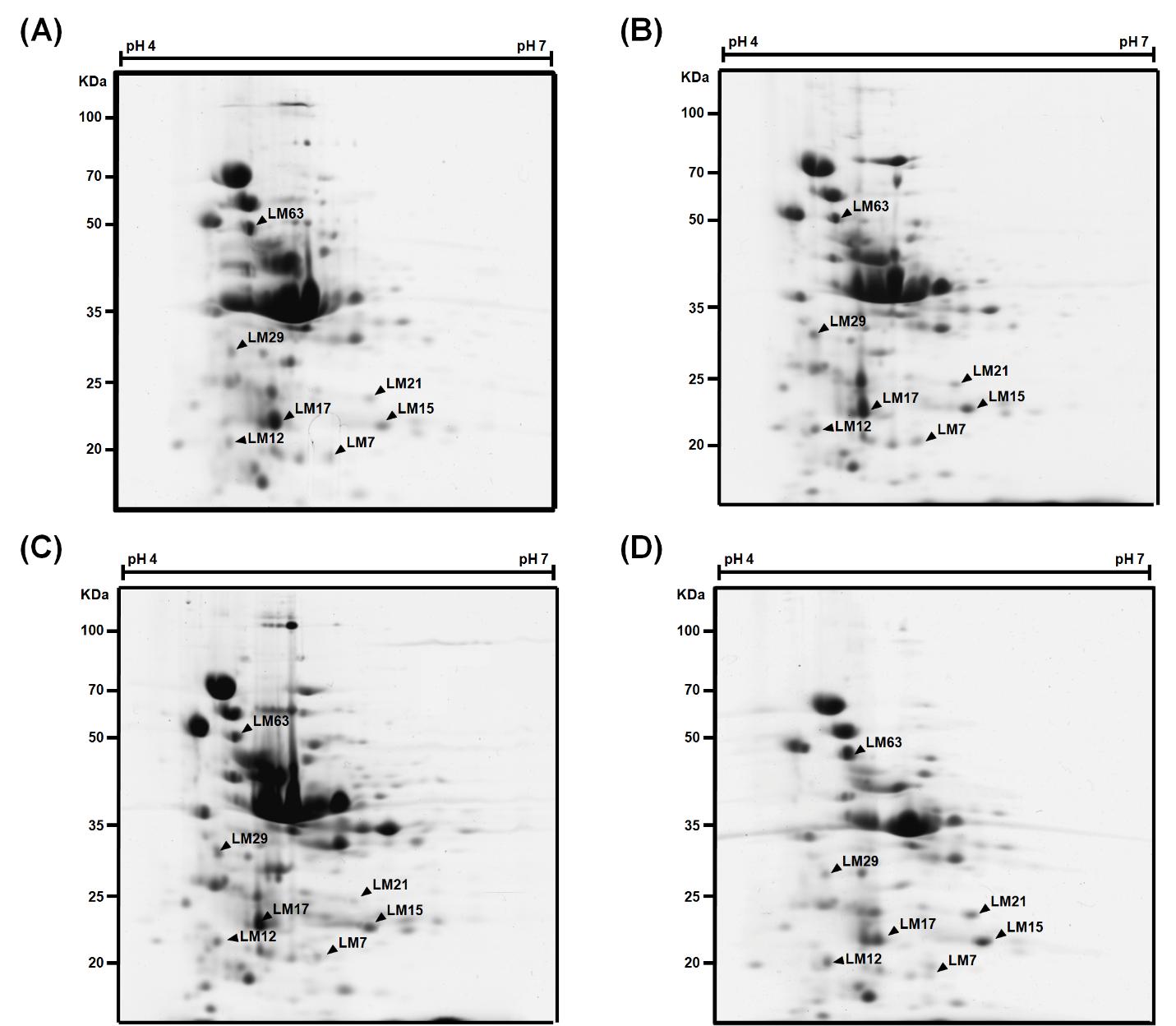

Supplement: Supplementary file 2 — Additional file 2: Fig. S2. 2DE gels of proteomes between wild type (A), LMS50 (B), LMS60 (C) and LMS70 (D). [file 13068_2016_662_MOESM2_ESM.docx]
